# Translation and initial validation of Chinese (Cantonese) version of Modified Fatigue Impact Scale (MFIS-C) in people with stroke

**DOI:** 10.1186/s12883-022-02832-w

**Published:** 2022-08-15

**Authors:** Shamay S. M. Ng, Tai Wa Liu, Joshua Tsoh

**Affiliations:** 1 Department of Rehabilitation Sciences, The Hong Kong Polytechnic University, Hung Hom, Hong Kong, Special Administrative Region of China; 2 School of Nursing and Health Studies, Hong Kong Metropolitan University, Ho Man Tin, Hong Kong, Special Administrative Region of China; 3 Department of Psychiatry, Prince of Wales Hospital & Shatin Hospital, Shatin, Hong Kong, Special Administrative Region of China

**Keywords:** Chronic stroke, Stroke rehabilitation, Fatigue, Modified fatigue impact scale

## Abstract

**Purpose:**

To translate and culturally adapt the Modified Fatigue Impact Scale (MFIS) to Chinese version, and to psychometrically test it in stroke population.

**Methods:**

This study consisted of 2 phases. In phase one, we translated and culturally adopted the original English version of MFIS into Chinese (Cantonese) (MFIS-C). In phase two, the MFIS was psychometrically tested using a cohort of community-dwelling people with stroke (*n* = 101) and healthy control (*n* = 50). Among the stroke participants, 52 of them were reassessed after a 1-week interval.

**Results:**

The MFIS-C demonstrated satisfactory content validity and good to excellent internal consistency. The overall MFIS-C and its subscales have good test-retest reliability. The MDC_95_ were 14.86, 7.49, and 9.70 for the overall MFIS-C physical, cognitive and psychosocial subscales, respectively. The overall MFIS-C and its 2 subscales have significant weak to moderate negative correlations with the Community Integration Measure and the 12-item Short Form Health Survey Version 2. Our findings revealed that the people with chronic stroke living in Hong Kong were reported to have high level of fatigue.

**Conclusion:**

The MFIS-C is a reliable and valid measure for assessing the level of fatigue in people with stroke.

**Supplementary Information:**

The online version contains supplementary material available at 10.1186/s12883-022-02832-w.

## Introduction

Fatigue refers to the difficulties in initiating or sustaining voluntary activities [1]. It is common among people with stroke and it affects 38 to 68% of the stroke population [2, 3]. Fatigue can be classified as non-pathological or pathological origin. While pathological fatigue is commonly found in the stroke population, along with certain chronicity from months to years and could not be relieved by rest [4], it could be further categorized into physical and mental domains based on difference in origin. Physical fatigue is more related to peripheral fatigue as it reflects the decline of muscle performance over time [1], whereas mental fatigue is considered a component of central fatigue and the decline in cognitive function of individuals [1]. Sometimes it might also be noted as the subjective perception of significant diminished energy level [5].

Severity of fatigue experienced by people with stroke was reported to be high [6], which affected the functional capacity of people with stroke [7]. Christensen et al. revealed that severity of fatigue experienced by people with stroke was 10.5% higher than healthy adults [6]. Indeed, results of recent studies demonstrated that increased level of fatigue was associated with increased dependency in activities of daily living [7], disability and burden of care [8] in people with stroke. Therefore, a quantitative representation capturing both the physical and mental facets of fatigue would be an invaluable tool for devising a more holistic treatment approach to improve the functional capacity of people with stroke.

Currently, the Fatigue Severity Scale (FSS) and the Fatigue Assessment Scale (FAS) are the two commonly used and validated fatigue scales in people with stroke [9, 10]. The FSS has excellent psychometric properties in people with stroke [9]. However, it is unidimensional and only focuses on physical fatigue [11]. The FAS is another fatigue scale with high test-retest reliability in people with stroke [10]. Similar with FSS, the FAS is unidimensional and has poor internal consistency in people with stroke [12, 13]. Moreover, while the FSS and FAS items were operationalized in non-specific conditions (E.g, FSS item 5 “Fatigue causes frequent problems for me”; FAS item 9 “Mentally, I feel exhausted”), the MFIS adopted more context-specific items to operationalize the construct of fatigue (E.g., MFIS item 8 “I have been less motivated to participate in social activities; MFIS item 15 “I have had trouble finishing tasks that require thinking”). Using context-specific items could minimize the subjectivity in interpreting the construct of fatigue represented by the items and enhanced the item and overall reliability of the MFIS.

To address the inadequacy of FAS and FSS, the Modified Fatigue Impact Scale (MFIS) could be a good alternative for measuring the level of fatigue in patients with stroke. The original FIS is a 40-item multidimensional scale comprised of physical, cognitive and psychosocial subscales [14]. It was shortened to the 21-item MFIS by the Multiple Sclerosis Council for the development of clinical practice guidelines. The psychometric properties of the MFIS have been tested in patients with various types of neurological disorder, including multiple sclerosis (MS) [14–16], Parkinson’s Disease (PD) [17] and traumatic brain injury (TBI) [18]. Using the PD and TBI populations, Schiehser et al. revealed the possibility of refining the factor structure from 3 to 2, including the cognitive subscale and physical and psychosocial subscales, which was theoretically consistent with the definition of fatigue [17, 19]. However, the MFIS has not been translated and culturally adapted to Chinese (Cantonese) version nor has it been validated for people with stroke. Therefore, the objectives of this study were to (1) translate and culturally adapt the English version of MFIS to Chinese (Cantonese) version (MFIS-C), (2) examine the psychometric properties of the MFIS-C, including the internal consistency, test-retest reliability, correlations between the MFIS-C with various stroke-specific health-related measures, and the minimally detectable change, and (3) the level of community-dwelling people with chronic stroke.

## Methodology

### Translation

The first phase of this study was the translation of the original English version of MFIS into a Chinese (Cantonese) pilot version of MFIS which was based on the standard forward- and backward translation guidelines [20]. First, the English version of MFIS was translated into Chinese (Cantonese) (forward translation) by two independent bilingual translators, one with rehabilitation training background, and the other one was a professional translator without rehabilitation training background. Two initial Chinese (Cantonese) drafts were produced independently (MFIS-C-D_1 and 2_). Second, the two initial Chinese (Cantonese) drafts (MFIS-C-D_1 and 2_) and the original English version of MFIS were reviewed by the two independent bilingual translators. Discrepancies found in the two drafts were discussed and resolved between them to reach a consensus Chinese (Cantonese) version (MFIS-C-D_3_). Third, the consensus version of MFIS-C (MFIS-C-D_C_) was then translated back into English (backward translation, MFIS-C-D_E_) by another two independent bilingual translators who were not involved in the forward translation process, including one with rehabilitation training background, and the other one was a professional translator without rehabilitation training background. Linguistic discrepancies were discussed and resolved among these two independent bilingual translators. In the fourth step, the MFIS-C-D_E_ was evaluated by an expert panel consisted of six panel members who have at least 6 years of experience in stroke rehabilitation practice and/or research, including three registered physiotherapists, one rehabilitation therapist, one registered nurse and one mental health nurse, on its equivalence of content, semantics, conceptual as well as technical discrepancies with the original English version of MFIS to produce the pilot version of Chinese (Cantonese) MFIS (MFIS-C-pilot). Then the MFIS-C-pilot was tested on 10 subjects with stroke to ensure its fluency, clarity and comprehensibility. The finalized version of MFIS-C was then established.

### Sample and data collection procedure

In phase 2 of the present study, convenient sampling was used to recruit 101 people with stroke and 50 healthy older people from a university-affiliated neurorehabilitation laboratory through poster advertisement. The inclusion criteria for stroke participants were: (1) chronic stroke with over 12 months post stroke with age 50 or older, (2) cognitively intact with score in the Abbreviated Mental Test ≥7, (3) capable of walking independently or with assistance or walking aids for 10 m, and (4) understand Cantonese. The subjects were excluded if they had: (1) unstable medical conditions such as coronary disease, comorbid psychiatric diseases, neurological problems (e.g. multiple sclerosis and Parkinson’s disease), and (2) musculoskeletal problems (e.g. painful knee osteoarthritis) that might impede the assessment procedures. The same inclusion and exclusion criteria were used for the recruitment of healthy older participants except for having a history of stroke.

We conducted all the assessments in a University-affiliated Neurorehabilitation Laboratory. Informed written consent was obtained from all the study participants prior to the study began. For the stroke participants, the sociodemographic data sheet, MFIS-C, Fugl-Meyer Assessment of Lower Extremity (FMA-LE), Timed Up & Go Test (TUG), the Chinese version of the Community Integration Measure (CIM-C) and the Chinese version of the 12-Item Short Form Health Survey Version 2 (SF-12v2-C) were administered to them on day 1. After a 1-week interval (day 2), 52 of the 101 stroke participants were randomly selected by drawing lots for re-assessment with the MFIS-C. For the healthy participants, the sociodemographic data sheet and MFIS-C were administered on day 1 only. All the assessments were conducted according to the principles of the Declaration of Helsinki for human experiments.

### Outcome measures

#### Modified fatigue impact scale (MFIS)

The Modified Fatigue Impact Scale (MFIS) is a shortened version of Fatigue Impact Scale (FIS) which was initially developed to assess the level of fatigue in people with multiple sclerosis (MS). The items are scored according to a 5-point Likert scale (0 = Never, and 1 = Seldom, 2 = Sometimes, 3 = Often, 4 = almost always) to assess the frequency of fatigue impact to their daily life in the past 4 weeks. According to Schiehser et al. the MFIS could be refined into a 2 factor structure measure with total score ranging from 0 to 84 and a higher score implies a greater impact of fatigue [17, 19]. The internal consistency of the MFIS was reported to be excellent (Cronbach’s alpha = 0.81–0.97) in people with MS [15, 16], Parkinson’s Disease [17] and traumatic brain injury [19]. The test-retest reliability of the MFIS was found to be good (ICC = 0.73–0.91) in people with MS [14, 15].

#### Fugl-Meyer assessment of lower extremity (FMA-LE)

The FMA-LE was used to assess the motor control of the lower limb [18]. It consists of items evaluating reflexes, synergic patterns and coordination, with a maximum score of 34 points. Each item is rated on a 3-point ordinal scale, in which 2 points, 1 point and 0 point correspond to complete performance, partial performance, and no performance respectively. The inter-tester reliability of the FMA-LE was found to be excellent (ICC = 0.92) on people with stroke [18].

#### Timed up & go test (TUG)

The TUG test was used to assess the basic functional performance in terms of mobility, balance and gait [21]. It consists of sitting, standing up, walking 3 m, turning 180°, then walking back and sitting down. The time for completion was recorded [21]. Shorter completion time indicates better functional capacity, balance, as well as faster gait speed [22]. The TUG test had a satisfactory intra-rater and interrater reliability with high ICC values of 0.95 [21].

#### Community integration measure (CIM)

The Chinese version of the Community Integration Measure (CIM-C) was used to examine the participants’ level of community integration. The questionnaire consists of 10 items which investigate different domains of community integration, such as belonging and independent participation. An unweighted single summary score from 10 to 50 would be obtained [23]. Moreover, excellent internal consistency (Cronbach’s alpha = 0.84) and test-retest reliability (ICC = 0.84) were observed in the CIM-C done in people with stroke [24].

#### The 12-item short form health survey version 2 (SF-12v2)

The Chinese version of the 12-Item Short Form Health Survey Version 2 (SF-12v2-C) was used to assess the participant’s health-related quality of life. The scale comprises 12 items and produces two summary scores (physical component score and mental component score) [25]. The physical component score and mental component score both ranges from 0 to 100. A higher score indicates a better health-related quality of life. The SF-12v2 demonstrated moderate to excellent internal consistency in all subscales (Cronbach’s α = 0.62–0.82) except the mental health subscale (Cronbach’s α = 0.48). It was reported to have good test-retest reliability (ICC = 0.60–0.82) [25].

### Data analysis

The item level CVI (I-CVI) was calculated by the number of panel members choosing grade 3 (quite relevant) or grade 4 (highly relevant) over the total number of panel members [26]. An I-CVI value of 1.0 is considered a quite or highly relevant item. The scale-level CVI (S-CVI) was determined by averaging the I-CVIs (scale-level content validity index, averaging calculation method; S-CVI-Ave) and calculating the proportion of item achieving a relevance rating of 3 or 4 by all the panel members (scale-level content validity index, universal agreement method; S-CVI-UA). A S-CVI value of 1.0 represents the acceptable content validity of the overall scale.

IBM SPSS Statistics 26 was used to conduct all statistical analysis. Statistical significance was taken at *p* < 0.05. Descriptive statistics were adopted to summarize the participants’ demographic characteristics and responses towards the MFIS-C. Internal consistency was determined using Cronbach’s alpha coefficient and item-total correlations. Test-retest reliability was estimated by intraclass correlation coefficient (ICC_3,1_) as the rater was fixed for the two testing occasions. ICCs of > 0.9, 0.75–0.90, 0.50–0.75 and < 0.50 indicate excellent, good, moderate and poor correlation, respectively [27]. Standard Error Measurement (SEM) was calculated by Baseline SD x √1- Intraclass coefficient [28]. Then, minimal detectable change (MDC) was found using the equation of SEM × 1.96 x √2 [28]. It indicates the minimal amount of change that can be interpreted as a real change in the scale.

Correlations of MFIS-C scores with FMA-LE scores, TUG completion times, CIM-C and SF-12v2-C scores were assessed by Pearson’s r or Spearman’s rho depending on the result of the K-S test, which was used for determining if the data are parametric or non-parametric. If the data are parametric, Pearson’s r would be used for correlation analysis. Otherwise, Spearman’s rho would be selected as the analysis method. Correlation coefficient ranges from 0.00 to 0.10 (negligible correlation), 0.10 to 0.39 (weak correlation), 0.40–0.69 (moderate correlation), 0.70–0.89 (strong correlation) and greater than 0.9 (very strong correlation) [29].

For the sample size calculation of the test-retest reliability, an estimated ICC value of 0.9 was adopted as no previous study assessing the test-retest reliability of the MFIS in people with stroke. With null hypothesis ICC value of 0.8 and a significance level of 0.05, the minimum required sample size was 46 to achieve 80% power to detect an ICC value of 0.9. The test-retest reliability sample size was estimated using the PASS software (Version 14NCSS, LLC. Kaysville, Utah, USA). For the sample size required for correlational analyses, significant weak correlations (r < 0.25) between the MFIS-C and other outcome measures were assumed. To achieve 80% power and a significant level of 0.05, the minimum sample size is 95.

## Results

### Content validity

All the I-CVI, S-CVI/Ave and S-CVI/UA values are 1, suggesting that the validity of each individual item and the overall MFIS-C are satisfactory (Appendix I, Supplementary file).

### Characteristics of the subjects

A total of 101 stroke participants recruited in this study and details of their characteristics were summarized in Table 1.

**Table 1:** Characteristics of participants

	Stroke participants (*n* = 101)	Healthy participants (*n* = 50)
Age (y), mean + SD	63.82 ± 6.40	61.78 ± 7.41
Sex, n (%)		
Women	43 (42.6)	35 (70.0)
Men	58 (57.4)	15 (30.0)
BMI (kg/m^2^), mean + SD	24.13 ± 3.02	22.43 ± 3.19
Education level, n (%)		
Primary or below	23 (22.8)	9 (18.0)
Secondary	64 (63.4)	33 (66.0)
College or above	14 (13.8)	8 (16.0)
Marital status, n (%)		
Single	9 (8.9)	4 (8.0)
Married	76 (75.2)	45 (90.0)
Divorced/separated	9 (8.9)	1 (2.0)
Widowed/widowered	7 (6.9)	0
Living arrangement, n (%)		
Alone	10 (9.9)	4 (8.0)
With others	91 (90.1)	46 (92.0)
Type of stroke, n (%)		
Ischemia	69 (68.3)	
Haemorrhage	32 (31.7)	
Years since stroke (y), mean + SD	6.74 ± 4.42	
Hemiplegic side, n (%)		
Left	46 (45.5)	
Right	55 (54.5)	
FMA-LE,, mean + SD	26.12 ± 4.46	
TUG, mean + SD	17.64 ± 14.23	
CIM-C, mean + SD	40.38 ± 7.05	
PCS, mean + SD	39.25 ± 9.09	
MCS, mean + SD	48.62 ± 10.64	

*n* number, *BMI* body mass index, *FMA-LE* Fugl-Meyer assessment for the lower extremities, *TUG* Timed Up & Go Test, *CIM-C* the Chinese version of Community Integration Measure, *PCS* physical component summary, *MCS* mental component summary

### Level of fatigue among Hong Kong Chinese people with stroke

The overall, cognitive, and physical and psychosocial subscales mean scores were 39.60 (SD 13.48), 16.85 (SD 6.36) and 22.75 (SD 8.21), respectively (Table 2). The item with the highest score (2.34 ± 0.99) was item 6 (I have had to pace myself in my physical activities), and the items with the lowest scores were item 3 (1.63 ± 1.02) (I have been unable to think clearly) and item 15 (1.63 ± 0.85) (I have had trouble finishing tasks that require thinking). Table 3 compares the MFIS-C subscale scores with different socio-demographics. Independent t – tests showed that there were no significant difference in both the MFIS-C subscale scores of stroke participants between men and women, living alone and others, ischemic and haemorrhagic stroke, and left or right side hemiplegia, while one-way ANOVA revealed that there were no significant differences between stroke participants with different educational status (primary, secondary and tertiary or above) and marital status (single, married, widowed/widowered, and divorced/separated). Healthy participants had lower overall (t = 3.94, *p* < 0.001), cognitive (t = 2.89, *p* = 0.005), and physical and psychosocial (t = 4.56, p < 0.001) subscales mean scores than those of stroke participants.

**Table 2:** Mean and standard deviation (SD), mode and median of MFIS-C item scores of stroke participants (*n* = 101)

Items.	Mean ± SD	Mode	Median
1	1.52 ± 0.90	2	2
2	1.76 ± 1.04	2	2
3	1.63 ± 1.02	2	2
4	1.68 ± 1.23	2	2
5	1.84 ± 1.08	2	2
6	2.34 ± 0.99	2	2
7	2.13 ± 1.01	2	2
8	2.03 ± 1.10	2	2
9	1.99 ± 1.20	3	2
10	2.10 ± 1.02	2	2
11	1.70 ± 0.99	2	2
12	1.70 ± 1.03	1	2
13	2.04 ± 1.06	2	2
14	1.79 ± 0.95	2	2
15	1.63 ± 0.85	2	2
16	1.65 ± 0.90	2	2
17	2.31 ± 1.00	2	2
18	1.66 ± 0.91	1	2
19	1.73 ± 0.99	2	2
20	2.25 ± 1.03	2	2
21	2.10 ± 1.21	2	2
Overall	39.60 ± 13.48		
Cognitive	16.85 ± 6.36		
Physical/psychosocial	22.75 ± 8.21		

MFIS-C, the Chinese (Cantonese) version of Modified Fatigue Impact Scale (MFIS)

**Table 3:** Comparisons for fatigue of stroke participants with different characteristics (*n* = 101)

	Overall MFIS	t, *F*-ratio (*p*-value)	Cognitive subscale, mean ± SD	t, *F*-ratio (p-value)	Physical/psychosoical subscale, mean ± SD	t, *F*-ratio (p-value)
Gender^a^		t = 0.20 (*p* = 0.84)		t = 0.47 (*p* = 0.64)		t = −0.04 (*p* = 0.97)
Male	39.84 ± 11.87		17.12 ± 5.53		22.72 ± 7.46	
Female	39.28 ± 15.53		16.49 ± 7.38		16.49 ± 7.38	
Educational status^b^		*F* = 0.07 (*p* = 0.94)		*F* = 0.49 (*p* = 0.62)		*F* = 0.03 (*p* = 0.97)
Primary	40.35 ± 9.88		17.61 ± 5.66		22.74 ± 6.18	
Secondary	39.23 ± 14.33		16.38 ± 6.47		22.86 ± 8.82	
Tertiary or above	40.07 ± 15.31		17.79 ± 7.11		22.29 ± 8.74	
Marital status^b^		*F* = 0.02 (*p* = 1.00)		*F* = 0.12 (*p* = 0.95)		*F* = 0.02 (*p* = 1.00)
Single	40.33 ± 19.43		17.56 ± 8.00		22.78 ± 11.72	
Married	39.41 ± 12.54		16.63 ± 5.90		22.78 ± 7.81	
Widowed/widowered	39.86 ± 17.81		17.33 ± 7.35		23.00 ± 7.37	
Divorced/separated	39.86 ± 17.81		17.71 ± 8.85		22.14 ± 9.26	
Living status^a^		t = 1.06 (*p* = 0.31)		t = 1.17 (*p* = 0.27)		t = 0.77 (*p* = 0.46)
Alone	44.00 ± 13.84		19.40 ± 7.35		24.60 ± 7.97	
With others	39.12 ± 13.43		16.57 ± 6.22		22.55 ± 8.25	
Type of stroke^a^		t = −0.90 (*p* = 0.93)		t = −0.22 (*p* = 0.82)		t = 0.03 (*p* = 0.98)
Ischemia	39.52 ± 12.53		16.75 ± 6.32		22.77 ± 7.52	
Haemorrhage	39.78 ± 15.54		17.06 ± 6.54		22.72 ± 9.67	
Hemiplegic side^a^		t = −0.72 (*p* = 0.48)		t = −1.29 (*p* = 0.20)		t = − 0.19 (*p* = 0.85)
Left	38.57 ± 12.22		15.98 ± 5.77		22.59 ± 7.49	
Right	40.47 ± 14.50		17.58 ± 6.77		22.89 ± 8.84	

MFIS-C, the Chinese version of Modified Fatigue Impact Scale

^a^independent t test

^b^one way ANOVA

### Internal consistency

The overall MFIS-C (Cronbach’s Alpha = 0.92) and both MFIS-C subscales demonstrated excellent and good internal consistency (Cognitive subscale, Cronbach’s Alpha = 0.85; physical and psychosocial subscale, Cronbach’s Alpha = 0.89), respectively (Table 4). There was moderate to good item-total correlation ranging from 0.46 (item 18) to 0.63 (item 3) and from 0.50 (item 4) to 0.71 (item 9) in the cognitive subscale and physical and psychosocial subscale, respectively. No item deletion could improve the values of Cronbach’s Alpha in either of the subscale.

**Table 4:** Internal consistency of MFIS-C (stroke participants, *n* = 101)

Item		Corrected item-total correlation	Cronbach’s 𝛼 if item deleted
Cognitive subscale			
1	I have been less alert	0.60	0.83
2	I have had difficulty paying attention for long periods of time.	0.55	0.84
3	I have been unable to think clearly.	0.63	0.83
5	I have been forgetful.	0.55	0.84
11	I have had difficulty making decisions.	0.55	0.84
12	I have been less motivated to do anything that requires thinking	0.51	0.84
15	I have had trouble finishing tasks that require thinking.	0.57	0.84
16	I have had difficulty organizing my thoughts when doing things at home or at work.	0.56	0.84
18	My thinking has been slowed down.	0.46	0.85
19	I have had trouble concentrating.	0.56	0.84
Physical/social subscale			
4	I have been clumsy and uncoordinated.	0.50	0.89
6	I have had to pace myself in my physical activities.	0.65	0.88
7	I have been less motivated to do anything that requires physical effort.	0.58	0.88
8	I have been less motivated to participate in social activities.	0.66	0.88
9	I have been limited in my ability to do things away from home.	0.71	0.88
10	I have had trouble maintaining physical effort for long periods.	0.64	0.88
13	My muscles have felt weak.	0.65	0.88
14	I have been physically uncomfortable.	0.57	0.89
17	I have been less able to complete tasks that require physical effort.	0.62	0.88
20	I have limited my physical activities.	0.63	0.88
21	I have needed to rest more often or for longer periods.	0.60	0.88
Overall MFIS, Cronbach’s alpha = 0.92			
Cognitive subscale (1,2, 3, 5, 11, 12, 15, 16, 18, 19), Cronbach’s alpha = 0.85			
Physical and psychosocial subscale (4, 6, 7, 8, 9, 10, 13, 14, 17, 20, 21), Cronbach’s alpha = 0.89			

### Test-retest reliability and MDC

Fifty-two of the stroke participants randomly selected by drawing lots for reassessment after a 1-week interval. The overall MFIS-C and MFIS-C subscales demonstrated good test-retest reliability (Table 5). The ICC values for the individual items ranged from 0.53 to 0.65, with item 11 (I have had difficulty making decisions) and 19 (I have had trouble concentrating) showing the least consistency, and item 3 (I have been unable to think clearly) and 5 (I have been forgetful) showing the most consistency. The MDC_95_ (SEM) and MDC_95%_ of the overall MFIS-C, cognitive subscale and physical and psychosocial subscale are 14.86 (5.38) and 38.3%, 7.49 (2.71) and 44.8%, and 9.70 (3.51) and 44.0%, respectively.

**Table 5:** The results of test-retest reliability

Item	Mean 1	Mean 2	ICC	95%CI low	95%CI high
1	1.40	1.63	0.64	0.43	0.78
2	1.67	1.71	0.56	0.34	0.72
3	1.52	1.63	0.65	0.46	0.78
4	1.69	1.81	0.62	0.42	0.76
5	1.79	1.77	0.65	0.47	0.79
6	2.31	2.10	0.58	0.37	0.73
7	2.21	2.27	0.57	0.35	0.73
8	2.17	1.85	0.64	0.41	0.78
9	2.06	2.23	0.59	0.39	0.74
10	2.15	2.21	0.61	0.40	0.76
11	1.81	1.79	0.53	0.30	0.70
12	1.77	1.83	0.57	0.35	0.73
13.	1.98	1.73	0.63	0.43	0.77
14.	1.69	1.67	0.59	0.38	0.74
15.	1.75	1.62	0.57	0.35	0.73
16.	1.71	1.60	0.55	0.32	0.71
17.	2.19	2.08	0.63	0.44	0.77
18.	1.63	1.52	0.57	0.35	0.73
19.	1.77	1.63	0.53	0.31	0.70
20.	2.23	2.21	0.56	0.34	0.72
21.	2.19	1.90	0.63	0.43	0.77
Cognitive subscale	16.83	16.73	0.83	0.72	0.90
Physical/social subscale	22.88	22.06	0.81	0.70	0.89
Overall	39.71	38.79	0.84	0.74	0.91

*ICC* intraclass correlation coefficient, *CI* confidence interval

### Correlation of MFIS-C scores

All the MFIS-C subscale scores have no significant correlations with the FMA-LE score and TUG completion time, but significant weak to moderate negative correlations with the CIM-C, PCS and MCS scores. (Table 6).

**Table 6:** Correlations between MFIS-C and other health measures

Measures	Overall MFIS-C	Cognitive subscale	Physical/social subscale
FMA-LE	*r* = −0.101, *p* = 0.313	*r* = 0.004, *p* = 0.97	*r* = −0.169, *p* = 0.091
TUG	*r* = 0.137, *p* = 0.175	*r* = 0.037, *p* = 0.714	*r* = 0.196, *p* = 0.051
CIM-C	*r* = − 0.357, *p* < 0.001	*r* = − 0.338, *p* = 0.001	*r* = − 0.325, *p* = 0.001
PCS	*r* = − 0.336, *p* = 0.001	*r* = − 0.243, *p* = 0.015	*r* = − 0.388, *p* < 0.001
MCS	*r* = − 0.346, *p* < 0.001	*r* = − 0.624, *p* < 0.001	*r* = − 0.539, *p* < 0.001

*FMA-LE* Fugl-Meyer Assessment for Lower Extremity, *TUG* Timed Up and Go Test, *CIM-C* the Chinese version of Community Integration Measure, *PCS* physical component summary, *MCS* mental component summary

## Discussion

This is the first study to translate and culturally adapt the Chinese (Cantonese) version of Modified Fatigue Impact Scale (MFIS-C) and validate its psychometric properties in Hong Kong Chinese people with stroke. The MFIS-C has satisfactory content validity, and the overall MFIS-C and its subscales have excellent and good internal consistency, respectively. Good test-retest reliability demonstrated in the overall MFIS-C and its subscales. Significant associations with measures of community integration and health-related quality of life were also identified. The level of fatigue of community-dwelling people with stroke was reported to be high in the present study, and we established the MDC values of 14.86, 7.49, and 9.70 for the MFIS-C physical, cognitive and psychosocial subscales, respectively, using this cohort of stroke participants.

The content validity of MFIS-C was assessed by the expert panel established in this study. The excellent item-level and scale-level content validity index supported the linguistic and equivalence of the MFIS-C in assessing the construct of fatigue among the community-dwelling Hong Kong Chinese people with chronic stroke.

Our findings revealed that the people with chronic stroke living in Hong Kong were reported to have high level of fatigue (39.60 ± 13.48). This finding was comparable to those with multiple sclerosis (MS) [28] which exceeds the cut-off score of 35.5–38 in people with MS [28, 30]. If the same MFIS cut-off score for people with MS is applied to our study, 60–71 subjects (59.41–70.29%) of our study will be considered as suffering from fatigue. The reasons of high level of fatigue reported among people with chronic stroke could possibly be explained by the underlying neurological conditions and mood disturbance after stroke. From the neuromuscular perspective, Kuppuswamy et al. suggested that decrease in corticomotor excitability in people with stroke was accompanied by suboptimal output from motor cortex, contributing to the decline in neural drive to alpha motor neurons [31]. Failure in signal propagation and excitation-contraction coupling were also identified in people with stroke [31]. From the psychological perspective, Wu et al. suggested that post-stroke fatigue was associated with post-stroke depression and lack of energy was one of the core symptoms of depression [32].

Among the MFIS-C items, the items with the highest item mean score were item 6 (I have had to pace myself during physical activity) with item mean score of 2.34 (0.99). This shows that physical fatigue was relatively more prominent in our subjects with stroke, affecting their daily physical tasks. The items with the lowest item mean score were item 3 (I have been unable to think clearly) with item mean score of 1.63 (1.02), and item 15 (I have had trouble finishing tasks that require thinking) also with an item mean score of 1.63 (0.85). The low scores in these 2 items could be attributed to the fact that our subjects were cognitively intact (AMT score > 7) and active community-dwelling stroke survivors who were eager to participate in social activities.

We calculated the item-level and subscale-level Cronbach’s alpha coefficients to examine the measuring ability of the MFIS-C to quantify the various facets of the construct fatigue. For the overall MFIS-C, our findings (Cronbach’s alpha = 0.92) were consistent with previous studies for various neurological populations, including Parkinson’s disease (PD) (Cronbach’s alpha = 0.95) [17] and traumatic brain injury (TBI) (Cronbach’s alpha = 0.95) [19] . All the items of the MFIS-C had item-total correlations of *r* > 0.30 (Field) supporting that they were measuring the same construct.

The present study demonstrated moderate to good test-retest reliability in the overall MFIS-C total score and all subscale scores with ICC of 0.81–0.84 when re-administered within a 1-week interval by the same tester. These findings were consistent with 2 previous studies that validated MFIS for patients with MS [14, 15] demonstrated good to excellent test-retest reliability with ICC of 0.73–0.93. The good reliability in our study could be due to three reasons: (1) accurate translation with relevant cultural adaptation, (2) optimal test-retest time interval of 1 week that minimize practice/memory effect as well as the chance of any occurrence of significant changes in the subjects’ life that may impact their self-perceived level of fatigue, and (3) the use of a standardized protocol with the same tester administrating the retest for the same subject to reduce inter-rater variability.

Among the 21 individual items of the MFIS-C, item 11 (I have had difficulty making decisions) and item 19 (I have had trouble concentrating) appeared to have comparatively low ICC of 0.53. One of the possible explanations may be that the term “decision making” and “concentrating” are too broad and vague and subject to participants’ subjective interpretation. In addition to the linguistic issues, another possible explanation is that both the item 11 and 19 operationalized the construct of fatigue in non-specific conditions instead of context-specific conditions. This might further reduce the reliability of these 2 items. To ensure the repeatability of clinical measures, it is advised to revisit the operationalization of the construct intended to measure to be easily quantifiable.

Minimally detectable change of MIF-C indicates the minimal change in score which reflects the real change in the result by eliminating the measurement errors. This is the first study to investigate MDC_95_ of MFIS-C in stroke survivors. The total and subscale’s MDC_95%_ ranged from 38.3 to 44% indicating that substantial random measurement error might be existed. This finding suggested that for clinical application, people with chronic stroke are required to achieve high change scores of the MFIS-C to represent a true change of level of fatigue.

Surprisingly, the overall and subscales of MFIS-C were found to have no significant correlation with a lower limb impairment measure (Fugl-Meyer Assessment for lower extremity, FMA-LE) and a functional mobility measure (Timed Up & Go Test, TUG) in the present study. One of the possible explanations for the insignificant correlation between the MFIS-C and FMA-LE was that the MFIS-C score is a subjective measurement of one’s perceived fatigue level during activities of daily living, while the FMA-LE is an objective measures of motor control of the lower limb. For community-dwelling people with chronic stroke, they could have been adapted to the post-stroke living style and adopted various compensatory strategies to accommodate the post-stroke lower limb impairment. Thus, no association was identified between this cohort of stroke participants. Moreover, the TUG is a useful tool in assessing fall risk and basic functional mobility in people with stroke [23] and requires only little energy consumption. Thus, functional tests with higher energy demand such as 6-minute walk test, which measures the total distance covered within 6 minutes at a tolerable and fast walking speed, might be required to establish a stronger correlation with level of fatigue.

As anticipated, the overall MFIS-C and MFIS-C subscales were found to have weak to moderate association with a community integration measure (Community Integration Measure, CIM) and a health-related quality of life measure (12-item Short Form Health Survey, SF-12v2). In a previous study of people with stroke [33], 78% respondents identified fatigue as the major hindrance of community reintegration. Our findings highlighted that fatigue could interfere the degree of social inclusion through various aspects, such as diminishing the motivation to participate in social activities and sense of being engaged in interpersonal relationships. These findings echoed a previous study examined community integration in people with MS, such that fatigue was associated with lower home competency which was important to community integration [34]. In addition, for mental well-being, a study showed that fatigue in people with MS was associated with depression, measured by MFIS and Beck Depression Inventory (BDI), with high correlation between all MFIS subscale scores and BDI (r = 0.59, 0.59, 0.49 respectively) [14]. This coincides with our findings that the MFIS-C scores significantly correlates with the SF-12v2 mental component score. Such significant correlations found in our study also agree with a study that investigated mental health in people with MS. In that study, fatigue in people with MS was evaluated to be significantly associated with poorer mental health, measured by mental index of Short Form 36, using linear regression analysis (β = − 0.23) [35].

This study has several limitations. In the present study, the translation of MFIS-C was suitable for the stroke population in Hong Kong because it was culturally adapted for the Hong Kong environment. However, perceptions of the MFIS-C in other Chinese populations such as Mainland China may be different given the difference in cultures. Second, the sample size calculation of our study was based on the reliability, which may not be sufficient to detect the significant correlations between MFIS-C scores and other outcome measures scores. Third, the findings of the present study could only be generalized to those fulfilling our inclusion and exclusion criteria. The study participants were not representative of those who experienced different level of post-stroke impairment and/or in different stage of stroke. Thus, the generalizability of the present study is limited. Fourth, our expert panel did not consist of a member with occupational training background. As occupational therapists have an important role in the assessment and rehabilitative interventions of people with fatigue syndrome, the inclusion of them might contribute to the assessment of cultural equivalence. Fifth, our stroke participants were not checked for history of chronic fatigue syndrome before entering the present study. Thus, it might or might not have impacts on our findings. Finally, we did not examine the construct validity of the MFIS-C due to the insufficient number of subject. Future study is recommended to examine the construct validity in order to expand the understanding of the psychometric properties of MFIS.

In conclusion, fatigue is a common complication after stroke and it may affect either or both the physical and mental health of people with stroke. The present study provides the evidence that MFIS is a valid and reliable measure which could be used to assess and monitor fatigue in both clinical and research settings. It can also help clinicians evaluate the effectiveness of rehabilitative interventions designed to alleviate fatigue in people with stroke. The identified MDC values could also help clinicians to assess the real change of level of fatigue among people with chronic stroke.

## Supplementary Information

**Additional file 1.**

## Data Availability

The datasets used and/or analysed during the current study are available from the corresponding author on reasonable request.

## Abbreviations

CIM: Community Integration Measure
CVI: Content validity index
FAS: Fatigue Assessment Scale
FSS: Fatigue Severity Scale
FMA-LE: Fugl-Meyer Assessment of Lower Extremity
ICC: Intraclass correlation coefficient
MDC: Minimal detectable change
MFIS: Modified Fatigue Impact Scale
MS: Multiple sclerosis
PD: Parkinson’s disease
SEM: Standard error measurement
SF-12v2: 12-Item Short Form Health Survey Version 2
TBI: Traumatic brain injury
TUG: Timed Up & Go Test
