# Supplementary material for: Translation and initial validation of Chinese (Cantonese) version of Modified Fatigue Impact Scale (MFIS-C) in people with stroke
Source: BMC Neurol. 2022 Aug 15;22:300. doi: 10.1186/s12883-022-02832-w (PMC9377082; doi:10.1186/s12883-022-02832-w)
Supplement: Supplementary file 1 — Additional file 1. [file 12883_2022_2832_MOESM1_ESM.docx]

Appendix I. Content validity index of the Chinese (Cantonese) version of Modified Fatigue Impact Scale

| Item | Expert A | Expert B | Expert C | Expert D | Expert E | | Expert F | Item-level content validity index |
| --- | --- | --- | --- | --- | --- | --- | --- | --- |
|  | 4 | 4 | 4 | 4 | 4 | | 4 | 1 |
|  | 4 | 4 | 4 | 4 | 4 | | 4 | 1 |
|  | 4 | 4 | 4 | 4 | 4 | | 4 | 1 |
|  | 4 | 4 | 4 | 4 | 4 | | 4 | 1 |
|  | 4 | 4 | 3 | 3 | 4 | | 4 | 1 |
|  | 4 | 4 | 4 | 4 | 4 | | 4 | 1 |
|  | 4 | 4 | 4 | 4 | 4 | | 4 | 1 |
|  | 4 | 4 | 4 | 4 | 4 | | 4 | 1 |
|  | 4 | 4 | 4 | 4 | 4 | | 4 | 1 |
|  | 4 | 4 | 4 | 4 | 4 | | 4 | 1 |
|  | 4 | 4 | 4 | 4 | 4 | | 4 | 1 |
|  | 4 | 4 | 4 | 4 | 4 | | 4 | 1 |
|  | 4 | 4 | 4 | 4 | 4 | | 4 | 1 |
|  | 4 | 4 | 4 | 4 | 4 | | 4 | 1 |
|  | 4 | 4 | 4 | 4 | 4 | | 4 | 1 |
|  | 4 | 4 | 4 | 4 | 4 | | 4 | 1 |
|  | 4 | 4 | 4 | 4 | 4 | | 4 | 1 |
|  | 4 | 4 | 4 | 4 | 4 | | 4 | 1 |
|  | 4 | 4 | 4 | 4 | 4 | | 4 | 1 |
|  | 4 | 4 | 4 | 4 | 4 | | 4 | 1 |
|  | 4 | 4 | 4 | 4 | 4 | | 4 | 1 |
| Scale-level content validity index/Mean | | | | | |  | | 1.0 |
| Total agreement | | | | | |  | | 21 |
| Scale-level content validity index/Universal agreement | | | | | |  | | 1.0 |
